# Detection of sexually transmitted infection and human papillomavirus in negative cytology by multiplex-PCR

**DOI:** 10.1186/1471-2334-10-284

**Published:** 2010-09-28

**Authors:** Hyo-Sub Shim, Songmi Noh, Ae-Ran Park, Young-Nam Lee, Jong-Kee Kim, Hyun-Jae Chung, Keum-Soon Kang, Nam Hoon Cho

**Affiliations:** 1 Department of Pathology, Yonsei University College of Medicine (Sungsanro 134), Seoul (120-752), South Korea; 2 Department of Affairs of Research Biostatistic, Yonsei University College of Medicine (Sungsanro 134), Seoul (120-752), South Korea; 3 Seegene Institute of Life Science (Taewon Bldg, 65-5), Seoul (138-050), South Korea; 4 Brain Korea 21 Project for Medical Science, Yonsei University (Sungsanro 134), Seoul (120-752), South Korea

## Abstract

**Background:**

The aim of this study was to determine the prevalence of human papillomavirus (HPV) and 15 species that cause sexually transmitted infections (STIs) in negative cytology. In addition, we compared the diagnostic performance of multiplex polymerase chain reaction (PCR) with widely available techniques used to detect HPV.

**Methods:**

We recruited 235 women of reproductive age who had negative cytology findings in a liquid-based cervical smear. STIs were identified by multiplex PCR, and HPV genotypes by multiplex PCR, hybrid capture 2, and DNA microaray; discordant results were analyzed by direct sequencing.

**Results:**

Approximately 96.6% of patients with negative cytology results were positive for pathogens that cause STIs. The pathogens most frequently detected were *Gardnerella vaginalis, Ureaplasma urealyticum*. The incidence of HPV in negative cytology was 23.3%. Low-risk HPV infection was significantly correlated with *Chalmaydia trachomatis*, and high-risk HPV infection was significantly correlated with *Group β streptococcus*. The analytical sensitivities of the multiplex PCR and DNA microarray were higher than 80%, and the analytical specificity was nearly 100% for all tests.

**Conclusions:**

Multiplex PCR yielded results that most of patients with negative cytology were positive for pathogens that cause STIs, and were more similar to that of DNA microarray, than that of hybrid capture 2 in terms of analytical sensitivity and prediction value of HPV infection.

## Background

Sexually transmitted diseases (STDs) are fairly common in people of reproductive age; more than 50% of new STD infections occur in adolescents. However, only 50% to 80% of reportable STDs that occur in the United States are actually reported [1]. Testing for STDs can result in negative cytology, that is, laboratory test results that revealed reactive cellular change (RCC) or atypical squamous cells of undetermined significance (ASCUS). RCC or ASCUS is routinely screened for, but they are not associated with neoplasms or preneoplastic stages known as squamous intraepithelial lesions (SILs). RCCs are cytological alterations related to inflammation or irritation but, whether subtle or marked, are typically not associated with human papillomavirus (HPV) infection. Common features of RCCs include the formation of small perinuclear halos, mild nuclear enlargement or nuclear degeneration, hyperchromasia, cytoplasmic pseudokeratinization, cytoplasmic vacuolization, and notably a background of inflammation. The abnormal cytology of ASCUS can usually be distinguished from RCC, but does not meet all criteria of HPV-infected koilocytes [2].

With the exception of a few organisms that cause sexually transmitted infections (STIs) such as *trichomoniasis *or *candidiasis*, most inflammatory agents are only rarely recognized under the microscope and usually produce nonspecific cytopathic effects. For example, bacterial vaginosis (BV) is a leading cause of abnormal vaginal discharge as a sequel of gynecologic conditions, including endometritis, pelvic inflammatory disease (PID), post-surgical abortion, post-hysterectomy infection, and cervical intraepithelial neoplasia [3]. However, few attempts have been made to identify the etiologic organisms of BV, and diagnoses have relied only on the presence of common "clue cells" detected in cervical swabs [4,5]. The prime manifestation of BV constitutes a massive micro-ecologic alteration of vaginal flora characterized by decreased *Lactobacillus spp*., logarithmically-increased *Gardnerella vaginalis*, and the presence of potentially pathogenic bacteria, including *Bacteroides, Peptococcus, Mobiliuncus*, *Ureaplasma urealyticum*, and *Mycoplasma spp*. *Mycoplasma spp*. and *U. urealyticum *are most frequently detected in the vaginal tract [6], and are strongly associated with infertility, intra-amniotic infection, postpartum infection, and pelvic inflammatory disease (PID) [1-5]. The multiple strains of *Mycoplasma *are the only cell wall-deficient, membrane-bound organisms capable of independent self-replication: they include *Mycoplasma hominis, M. genitalium, M. primatum, M. spermatophilum*, and *M. penetrans *[7,8].

Despite the fact that many pathogens are not detected by Gram stain, Gram-stained vaginal secretions are still accepted as the diagnostic gold-standard, rather than the culture of secretions. With the advent of advanced techniques such as those based on liquid-based cytology [9-13], it is now feasible to accurately and efficiently identify microorganisms in cervicovaginal swab specimens of asymptomatic patients with multiplex PCR or DNA chip. Although detection of infectious organisms is not the primary goal of cervical cytology, it can be an important component of screening in asymptomatic patients or subtle disease. We aimed to estimate the incidence of STIs in healthy women of reproductive age with negative cytologic findings by multiplex PCR and find any correlation of HPV infection with specific pathogens.

High-throughput technology including hybrid capture 2 (HC2) and DNA microarray has been currently adopted to detect HPV [14]. HC2 is a relatively easy method with high screening power, but it is unable to genotype or excludes low-risk HPV types. DNA microarray can identify specific genotypes, and can also detect co-infection in a single sample, but is labor-intensive. Despite widely available high-throughput technology for the detection of microbes or HPV, few studies of STDs and HPV with high fidelity data exist due to lack of a gold standard. We therefore designed multiplex PCR assays to compare with the available methods of HC2 and HPV DNA microarray, and analyzed discordant results by direct sequencing.

## Methods

A total of 235 married and sexually active women (mean age of 37.9 y; ages range 21 to 48 years) were recruited by the Department of cytopathology of Yonsei University College of Medicine for this study. Specimens collected from cervical liquid-based cytology medium were negative for intraepithelial lesions. Participants had no clinical STD symptoms that required a clinical visit, no history of STDs, and no abnormal Papanicolau tests (Pap smear) results. We also excluded patients with SIL or carcinoma, postmenopausal women, or those with previous operative or therapeutic history related to gynecologic disease. The study protocol was approved by the local institutional review board, and written informed consent was obtained from all participants.

### Multiplex-PCR for STI

#### Pretreatment and DNA Extraction of cervicovaginal swab specimens

Cervix swab samples were collected from the posterior fornix and lavaged with 5 mL of sterile phosphate-buffered saline (PBS pH 7.4). PreservCyt specimens were tested by Seeplex^® ^STI Master ACE Detection (Seegene, Seoul, Korea) or Seeplex^® ^HPV4A ACE Screening (Seegene) within 1 month of collection. PreservCyt transport medium containing endocervical cells was vortexed vigorously, and 1-mL samples were transferred into 1.5-mL polypropylene tubes, each containing 1 mL of PreservCyt transport medium. The tubes were centrifuged at 13,000 × *g *for 15 min at 20°C. Supernatants were discarded, and each cellular pellet was suspended in 200 μL of PBS. DNA was purified from these samples using the QIAamp DNA Mini Kit according to the manufacturers' instructions (Qiagen, Hilden, Germany). The quality and quantity of purified DNA was measured by spectrophotometry.

#### Multiplex PCR

Four primer sets were tested by STI multiplex PCR: Seeplex^® ^STI Master Panel 1, 2, 3, and HPV4A ACE Screening. Panel 1 comprised of *Neisseria gonorrhoeae (NG)*, *Mycoplasma hominis (MH), Chlamydia trachomatis (CT)*, *Ureaplasma urealyticum (UU)*, and *M. genitalium (MG)*. Panel 2 comprised *Trichomonas vaginalis (TV), Gardenerella vaginalis (GV), Bacteroides fragilis (BF), Mobiluncus curtisii (MC)*, and *M. mulieris (MM)*. Panel 3 comprised *Candida glabrata (CG), C. tropicalis (CT1), C. parapsilosis (CP)*, *Group β- streptococcus *(*GBS*), and *C. albicans (CA)*. Primers were designed such amplicon sizes differed sufficiently to be distinguished from each other; they ranged from 212 bp (*UU*) to 635 bp (*CG*) and the internal control was 981 bp. Optimized multiplex PCR was performed in 20 μL reactions containing DNA template, primer mixture (final concentration of each primer, 3 pmole), 2× Master mix (Seegene, Korea) and 30 μg/mL of 8-methoxypsoralen (MOP), which prevents contaminating DNAs from being amplified. PCR amplification was performed in an Applied Biosystem 9700 thermal cycler (Perkin-Elmer, Boston, MA, USA) with the following conditions: 94°C for 15 min, followed by 40 cycles of 94°C for 30 sec, 63°C for 1.5 min (or 60 °C for 1.5 min for HPV4A ACE Screening), and 72°C for 1.5 min; and final extension at 72 °C for 10 min. A DNA plasmid was added to the PCR reaction mixtures as an internal control to be co-amplified with the target DNAs collected from the clinical specimens. Sterile deionized water was included as a negative control in each batch of PCR reactions. Target organisms, target genes, accession numbers, and expected amplicon sizes are summarized in Additional File 1.

#### Multiplex-PCR-

HPV was detected with the Seeplex^® ^HPV4A ACE screening kit for 14 HR-HPV types (31, 33, 35, 39, 45, 51, 52, 56, 58, 59, 66, 67, 68, and 70) and 5 LR-HPV-types (6, 11, 42, 43 and 44). In addition individual genotyping of HPV-16 and HPV-18 was performed with different multiplex primer mixtures.

#### Hybrid capture 2 (HC2)

HC2 HPV test (Digene, Gaithersburg, MD) was performed according to the manufacturer's instructions to detect HPV, starting from 4 mL of liquid cytology specimens. The HC2-13 HR probe cocktail detects HPV types-16, 18, 31, 33, 35, 39, 45, 51, 52, 56, 58, 59, and 68. Relative light unit/cut-off ratio between 1 and 2.5 relative light units was used as the threshold for a positive result.

#### HPV Microarray

We used an HPV genotyping DNA microarray (Biocore Ltd, Korea, Seoul) with multiple oligonucleotide probes of L1 sequence of 26 types of HPV (HR: 16,18, 26, 31, 33, 35, 39, 45, 51, 52, 56, 58, 59, 66, 68, 69, 73 and 53; LR: 70, 6, 11, 32, 34, 40, 42, 43, 44, 54, 55, 57, 61 and 62). L1 consensus PCR products were hybridized to the probes on the microarray, and HPV genotypes were identified with a fluorescence scanner (GenPix 4000B, Axon Instruments Inc, CA) with a 532 nm laser for excitation of Cy3.

#### DNA sequencing and cloning

Discrepant results among the HC2, DNA microarray, and Seeplex^® ^HPV4A ACE screening kit results were resolved by sequencing with the PGMY09/PGMY11 primer sets. PCR reactions (20 μL) were performed with 2 μL template DNA, 1 μM of each primer, and 2x Master mix (Seegene, Korea). PCR amplification was carried out with an Applied Biosystem 9700 thermal cycler (Perkin-Elmer) with the following parameters: 94°C for 15 min followed by 40 cycles of 94°C for 30 sec, 55°C for 1 min, and 72°C for 1 min, and a final extension at 72°C for 10 min. The entire volume of each PCR reaction was run on a 2% agarose/1× Tris-acetate-EDTA gel containing ethidium bromide. The 465-bp bands amplified by the PGMY09/PGMY11 primers were excised and transferred to 1.5-mL tubes. DNA was extracted with the QiaexII gel extraction system (Qiagen, Valencia, CA) and eluted with 20 μL Tris-EDTA buffer. The products were ligated into the pGEM-T Easy vector (Promega, Madison, WI), transformed into chemically-competent JM109 cells, and plated onto two Luria-Bertani (LB) plates containing ampicillin, isopropyl-D-thiogalactopyranoside, and 5-bromo-4-chloro-3-indolyl-D-galactopyranoside. DNA was extracted from the white colonies with the Qiaprep Spin kit (Qiagen) and sequenced with the ABI Big Dye Terminator v3.1 cycle sequencing kit and an ABI 377 sequencer (Applied Biosystems, Foster City, CA). Sequences were trimmed to exclude the amplification primer and vector sequences, and the resulting fragments were analyzed by BLAST http://www.ncbi.nlm.nih.gov/blast. The top hit for each sequence was listed as the genotype.

### Statistical Analysis

The agreement rate of kappa value with respect to a quantitative measure of agreement between assay methods was calculated. The sensitivity of the HPV multiplex PCR, HC2 or DNA microarray relative to DNA direct sequencing is the proportion of positive samples in each assay among sequencing positive samples, and the specificity is the proportion of negative samples in each assay among those negative samples in direct sequencing. The positive predictive value is the proportion of positive samples in DNA direct sequencing, among those positive samples in each assay, and the negative predictive value is the proportion of negative samples in sequencing among those negative in each assay.

## Results

### Prevalence of STIs

**Figure 1:**
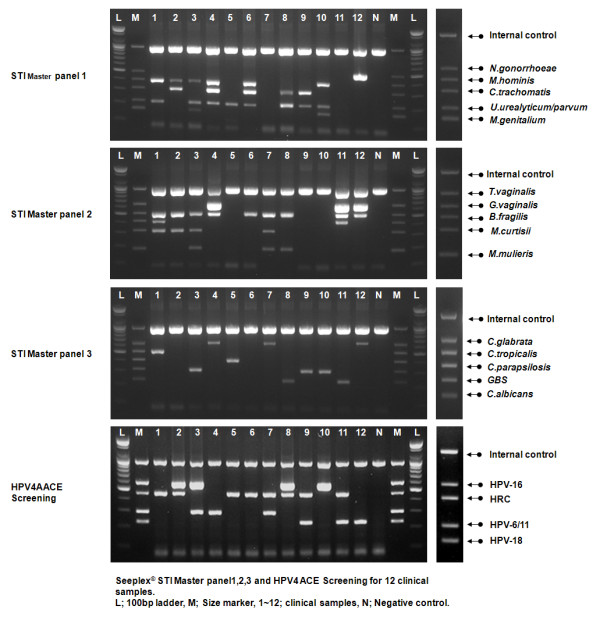
**Sexually transmitted infection and human papillomavirus (HPV) multiplex PCR**. Representative data of multiplex PCR with four multiplex primer mixes: Seeplex^® ^STI Master Panel 1 (MP1), 2 (MP2), 3 (MP3) and HPV4A ACE Screening. Panel 1 (B31) primers were designed so that amplicons were different sizes: 214 bp (*NG*), 253 bp (*MG*), 348 bp (*CT1*), 435 bp (*UU*), 502 bp (*MH*), and 800 bp (internal control). Panel 2 (B32) amplicons were 155 bp (*MM*), 197 bp (*MC*), 343 bp (*BF*), 509 bp (*GV*), and 647 bp (*TV*).

The overall prevalence of STIs in cervicovaginal samples of negative cytology, which was not clinically presented with BV, was 97.9% (230/235). Most patients of 84.9% (202/230) were co-infected by more than two microbes, whereas twenty-eight specimens were infected by a single agent (Fig. 1). Table 1 is an example to interpret data shown in Fig. 1 Most common agents we detected were *GV *found in 88.1% of the samples (207/235), *UU *in 63.0% (148/235), and *GBS *in 26.4% (62/235). The most frequently co-infected organism was observed in *MH *infections, which was commonly observed with *UU *(p = 0.000), *MM *(p = 0.000), *MC *(p = 0.000), and *CT *(p = 0.009). In addition, *GV *and *MC *(p = 0.014), *MC *and *MM *(p = 0.003), *MG *and *CA *(p = 0.006), and *TV *and *GBS *(p = 0.011) were similarly detected in the same samples.

**Table 1:** Interpretation of data shown in Figure 1.

Sample #	MP1	MP2	MP3	HPV4AACE
1	*M. hominis, U urealyticum/parvum*	*G. vaginalis, B fragilis*, *M. curtisii*	*C. tropicalis*	HRC

2	*M. hominis, C. trachomatis*	*G. vaginalis, M. curtisii*	*-*	HPV-16

3	*M. hominis, U urealyticum/parvum, M. genitlaium*	*G. vaginalis, M. curtisii*, *M. mulieris*	*GBS*	HPV-16

4	*M. hominis, C. trachomatis*, *U urealyticum/parvum*	*T. vaginalis, G. valginalis*	*C. glabrata*	HPV-16, HPV 6/11

5	*U urealyticum/parvum*	*-*	*C. parapsilosis*	HRC

6	*M. hominis, C. trachomatis*, *U urealyticum/parvum*	*G. vaginalis*	*-*	HRC

7	*-*	*G. vaginalis, M. curtisii*, *M. mulieris*	*C. glabrata*	HRC, HPV-6/11

8	*C. trachomatis, U urealyticum/parvum*	*G. vaginalis, M. mulieris*	*C. albicans*	HPV-16, HRC

9	*C. trachomatis, U urealyticum/parvum*	*-*	*GBS*	HRC, HPV-18

10	*M. hominis, U urealyticum/parvum, M. genitalium*	*-*	*GBS*	HPV-16

11	*-*	*T. vaginalis, G. vaginalis*, *B. fragilis*	*C. albicans*	HRC, HPV-18

12	*N. gonorrhoeae*	*T. vaginalis, G. vaginalis*	*C. glabrata*	HPV-18

HRC: HPV high-risk common 11 types

### HPV infection status

The incidence of HPV in negative cytology varied according to the assay used: multiplex-PCR, 24.7% (58/235); HC2, 21.3% (50/235); HPV chip, 22.6% (53/235). The HPV types which were most often detected included HPV-16 (PCR, 20.7%; DNA Chip, 26.4%) and HPV-18 (PCR, 8.6%; DNA chip, 11.3%). Less commonly detected HPV types included 51, 58, and 33 in the HR group, and 70 and 6 in the LR group. Eighteen LR-HPV types were detected by PCR (31.0%), eight by DNA microarray (15.1%), and 11 by direct sequencing. Infection with multiple genotypes was detected in 13 specimens (22.3%) by PCR and 14 specimens (26.4%) by DNA microarray.

### Comparison between HPV Multiplex PCR, HC2, and Microarray

Overall, there are good agreements between methods to detect HPV, and multiplex PCR showed the highest agreement ratio with relation to direct sequencing (PCR vs. sequence, Kappa = 0.8558; HC2 vs. sequence, Kappa = 0.8156; Chip vs. sequence, Kappa = 0.8539; PCR vs. HC2, Kappa = 0.7179; PCR vs. Chip, Kappa = 0.8082; HC2 with chip, Kappa = 0.7915;).

Because HC2 detects only HR group, 11 specimens with LR-HPV infection were excluded in the analytic sensitivity/specificity. The results of 25 of 235 specimens (10.6%) were discordant among methods; therefore these specimens were analyzed by direct sequencing. DNA chip produced more false negative results than HPV multiplex PCR and HC2 did (Table 2).

**Table 2:** Comparison of PCR, HC2, and Chip methods in 25 discordant samples.

	PCR	HC2	Chip	Sequence	False +	False -
1	LR	N	N	N	PCR	

3	LR	N**	N	70	PCR ^1^*	Chip

4	N	HR	N	53	HC2 ^2^*	PCR/Chip

5	HR	HR	N	66	HC2 ^3^*	Chip

6	HR	HR	N	54	PCR/HC2	Chip

7	LR	HR	N	11	HC2	Chip

8	N	HR	OTHER	83	HC2 ^4^*	PCR ^4^*

9	N	HR	OTHER	52	Chip: mistyping	PCR

10	N	HR	OTHER	N	HC2/Chip	

11	N	HR	N	39		PCR/Chip

12	HR	N**	70	70	PCR	

13	N	HR	16	16		PCR

14	HR	N	51	51		HC2

15	HR	N**	11	11	PCR	

16	HR	N**	70	70	PCR	

17	N	HR	16+39	16+39		HC2

18	N	HR	54	54	HC2 ^5^*	PCR ^5^*

19	N	HR	N	N		HC2

20	HR	HR	70	70	PCR	HC2

21	N	HR	N	N	HC2	

22	N	HR	other	N	HC2/Chip	

23	18+HR	N	18+70	18+70		HC2

24	HR+LR	N (equivocal)	73+43	73+43		HC2

25	HR	N	51	51		HC2

PCR (20 types; 18 HR types+2 LR types): 16, 18, 26, 31, 33, 35, **39**, 45, 51, **52**, **53**, 56, **58**, 59, **66**, 68, 73, and 82 + 6 and **11**

HC2: (13 HR types) 16,18,31,33,35,**39**,45,51,**52**,56,58,59, and 68

Chip (26 types; 18 HR types+8 LR types): 16,18, 26, 31, 33, 35, **39**, 45, 51, **52**, 56, **58**, 59, **66**, 68, 69, 73, 53 and LR: **70**, 6, **11**, 32, 34, 40, 42, 43, 44, **54**, 55, 57, 61, 62

1*: PCR LR does not cover HPV-70 type.

2*: HC2 HR does not cover HPV-53 type.

3*: HC2 HR does not cover HPV-66 type.

4*: HC2 and PCR PR do not cover HPV-83 type.

5*: PCR LR does not cover HPV-54 type.

6*: PCR LR does not cover HPV-67 type.

**: HR2 was not considered in this analysis because HR2 excludes LR HPV types.

**Figure 2:**
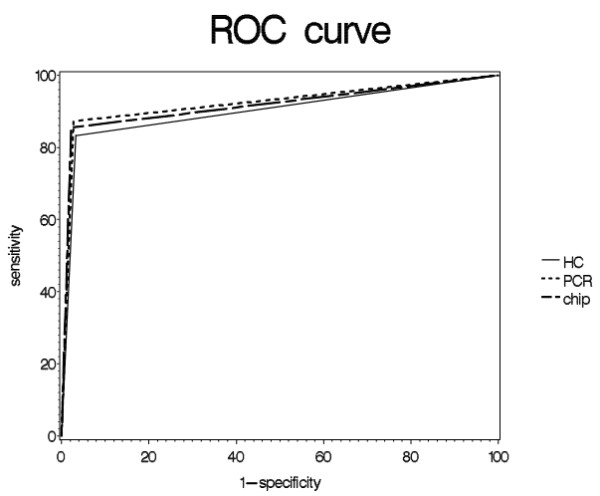
**ROC curve to detect HPV using multiplex-PCR, HC2, and DNA microarray**. ROC curve areas and 95% confidence intervals between each of the three different methods are shown.

Performance characteristics of the diagnostic test are shown in Table 3 . Positive predictive values associated with multiplex PCR and microarray were greater than 90%; the negative predictive value and accuracy rates were greater than 95%. HPV multiplex PCR and DNA microarray methods showed analytical sensitivities greater than 85%, and analytical specificity was nearly 100% with all methods. The area under the curve (AUC) was the highest for multiplex PCR at 0.922. Receiver operating characteristics (ROC) are shown in Fig. 2. However, there were no statistically significant differences between the three methods tested.

**Table 3:** Efficacy of multiplex-HPV PCR, HC2, and Chip, based on direct sequencing.

	True Positive	True Negative	False Positive	False Negative		HPV-all- Negative	
**PCR**	48	175	5	7		235	

**HC2**	45	173	6	9		233	

**Chip**	47	176	4	8		235	

	**Sensitivity**	**Sensitivity-l**	**Sensitivity-u**	**Specificity**	**Specificity-l**	**Specificity-u**	**AUC**

**PCR**	87.2727	78.4646	96.0808	97.2222	94.8214	99.6230	0.922

**HC2**	83.3333	73.3932	93.2735	96.6480	94.0113	99.2848	0.900

**Chip**	85.4545	76.1369	94.7722	97.7778	95.6243	99.9312	0.916

	**PPV**	**PPV-l**	**PPV-u**	**NPV**	**NPV-l**	**NPV-u**	**Accuracy**

**PCR**	90.5660	82.6965	98.4356	96.1538	93.3599	98.9478	94.8936

**HC2**	88.2353	79.3926	97.0779	95.0549	91.9051	98.2048	93.5622

**Chip**	92.1569	84.7782	99.5356	95.6522	92.7055	98.5988	94.8936

-l: lower limit of 95% confidence interval, -u: upper limit of 95% confidence interval.

AUC: area under the curve

PPV: positive predictive value,

NPV: negative predictive value

### Analysis of the correlation between STIs and HPV infection

Specimens positive for LR-HPV infection were frequently co-infected with *C. trachomatis *(4/18 HPV LR+ [22.2%]; p = 0.049), whereas those with HR-HPV were frequently detected with *GBS *(6/37 HPV HR+ [16.2%]; p = 0.016).

## Discussion

Reports of the incidence of STIs in asymptomatic women are not currently available, because of few attempts to recognize STI species despite advanced techniques based on liquid-based cytology [9-13].

We found that the overwhelming majority of patients with negative cytologic findings were positive for at least one STI (97.9%); many were positive for multiple infectious agents (84.9%). These results are surprising in that we did not observe high rates of infection with microbes that are typically widespread, such as *N. gonorrhea *and *C.trachomatis *[10,13]. In more than 25% of the samples, we detected microbes that cause asymptomatic, dormant non-gonococcal and non-chlamydial infections, such as *G. vaginalis, U. urealyticum, Group β streptococcus*, and *M. hominis*. Microbes detected in less than 2% of the specimens were *CT-1, NG*, and *C. parapsilosis (CP) *[13].

*MH *was often detected in the same sample with other microbes, such as *UU, M. mulieris (MM), M. curtisii (MC)*, and *CT*. However, co-infection of multiple mycoplasma strains has rarely been reported [15].

Because HPV and STIs rely on the same route of infection, this increased risk of HPV infection in patients with STIs was expected. Studies of women visiting STD clinics have reported a greater prevalence of HPV infection in this group than in the general population [16,17]. In one study, approximately half of the women evaluated at an STD clinic were infected with genital HPV, including HPV types-16, 83, 56, 52, and 59 (HR group) and 66 (LR group) [16]. In the present study of 235 asymptomatic women who had negative cytology results, we found that approximately 20% were infected with HPV. Using a genotyping microarray or grouping multiplex PCR, the most common HR types we detected were HPV types-16, 18, 51, 58, and 33, and the most common LR type was HPV-70. Epidemiologic data concerning HPV-types differ based on the method of detection used and the target samples. Multiple infections with more than two different HPV genotypes were found in 22% to 26% of the specimens, and HPV-16 was most detected (50%).

Specimens infected with LR-HPV were frequently co-infected with *CT*, whereas those with HR-HPV were coinfected with *GBS*. The fact that *CT *associates with LR-HPV rather than HR-HPV suggests that CT is less likely to contribute to cervical intraepithelial neoplasia or squamous cell carcinoma. Previous reports suggested that coinfection with *CT *and HPV is common in ASCUS and could contribute to the development of intraepithelial lesions [18-24]. It is possible that the modulation of cervical immune responses in response to *CT *may influence the clearance of HPV lesions [18,19], and suggesting that *CT *infection with HPV persistence is not a coincidence [20]. Likewise, we are the first to document the prevalence of coinfection of *GBS *and HR-HPV in negative cytology (including RCC and ASCUS). However, it remains uncertain that GBS may be an independent factor or cofactor for HPV in the development of cervical intraepithelial neoplasm. For identification of its cofactor, novel immune evasion strategies and the analysis of their functions in the context of viral and bacterial infections should be required in further studies.

We compared high-throughput HPV detection methods in the present study, and found that discrepancies with direct sequencing were observed only in 10.6%. The microarray (DNA chip) method also resulted in a significant number of false negative results. Analysis of 25discordant samples showed that multiplex PCR-HPV method exhibited the highest sensitivity, positive predictive value, and accuracy. However, each HPV detection method covered different genotypes, and different typing or grouping methods were used to present the data. For example, the relatively low sensitivity of HC2 may be due to its inability to detect LR genotypes, and its lower positive predictive values and accuracy may be due to limitations in detecting individual genotypes, and thus, multiple infections.

## Conclusions

In conclusion, asymptomatic women of reproductive age with negative cytologic findings had a high frequency of infection with *GV, UU, GBS*, and *MH*. Most HPV infections were not associated with any specific STI pathogen; however, LR-HPV infection correlated with *CT *infection, and HR-HPV with *GBS *infection. In terms of sensitivity and prediction value, multiplex PCR yielded data most similar to that of DNA microarray.

## Competing interests

The authors declare that they have no competing interests.

## Authors contribution

**HSS **and **SMN **summarized, analyzed data and equally drafted entire manuscript. **ARP **performed statistical analysis of given data. **YNL & KSK **participated in cytology screening and sample preparation with performance of HC2/DNA microarray. **JKK & HJC **performed multiplex PCR. **NHC **designed and participated in DNA direct sequencing, and revised manuscript. **All authors read and approved the final manuscript**.

## Short Summary

Two hundred thirty-eight asymptomatic women of reproductive age with negative cytology had high frequency of sexually-transmitted infection (96.6%), and 24.4% had HPV.

## Pre-publication history

The pre-publication history for this paper can be accessed here:

http://www.biomedcentral.com/1471-2334/10/284/prepub

## Supplementary Material

Additional file 1 **Summarized data of target organisms, target genes, accession numbers, and the expected amplicon sizes**. Primers were designed such amplicon sizes differed sufficiently to be distinguished from each other; they ranged from 212 bp (*UU*) to 635 bp (*CG*) and the internal control was 981 bp. Click here for file
