# Supplementary material for: Detection of sexually transmitted infection and human papillomavirus in negative cytology by multiplex-PCR
Source: BMC Infect Dis. 2010 Sep 28;10:284. doi: 10.1186/1471-2334-10-284 (PMC2956726; doi:10.1186/1471-2334-10-284)
Supplement: Additional file 1 — Summarized data of target organisms, target genes, accession numbers, and the expected amplicon sizes. Primers were designed such amplicon sizes differed sufficiently to be distinguished from each other; they ranged from 212 bp (UU) to 635 bp (CG) and the internal control was 981 bp. [file 1471-2334-10-284-S1.DOCX]

**Additional File 1.** Summarized data of target organisms, target genes, accession numbers, and the expected amplicon sizes.

| **Organism** |  | **Target DNA** |  | **Accession No.** |  | **Length (bp)** |
| --- | --- | --- | --- | --- | --- | --- |
| Internal control |  | CESA3 |  | NM_120599 |  | 981 |
| **Panel 1**  *N.gonorrhoeae* |  | porA pseudogene |  | AJ223447 |  | 495 |
| *M.hominis* |  | Gap |  | AJ243692 |  | 398 |
| *C.trachomatis* |  | Cryptic plasmid |  | M19487 |  | 314 |
| *U.urealyticum* |  | urease |  | AF085729 |  | 212 |
| *U.parvum* |  | urease |  | AF085733 |  | 212 |
| **Panel 2** |  |  |  |  |  |  |
| *T.vaginalis* |  | actin |  | U63122 |  | 647 |
| *G.vaginalis* |  | ITS |  | L08167 |  | 509 |
| *B.fragilis* |  | gyrB |  | AP006841 |  | 415 |
| *M.curtisii* |  | Cpn60 |  | AY123679 |  | 320 |
| *M.mulieris* |  | ITS |  | AF385928 |  | 180 |
| **Panel 3**  *C.glabrata* |  | RPR1 |  | EF526211 |  | 635 |
| *C.parapsilosis* |  | top2 |  | AB049114 |  | 409 |
| GBS |  | cfb |  | X72754 |  | 314 |
| *C.albicans* |  | phr1 |  | M90812 |  | 234 |
